# A Perspective on the Integration of eHealth in Treatment of Offenders: Combining Technology and the Risk-Need-Responsivity Model

**DOI:** 10.3389/fpsyt.2021.703043

**Published:** 2021-09-01

**Authors:** Hanneke Kip, Yvonne H. A. Bouman

**Affiliations:** ^1^Department of Psychology, Health and Technology, University of Twente, Enschede, Netherlands; ^2^Department of Research, Stichting Transfore, Deventer, Netherlands

**Keywords:** forensic psychiatry, offenders, eHealth, technology, RNR-model, risk factors, responsivity

## Abstract

While there are multiple ways in which eHealth interventions such as online modules, apps and virtual reality can improve forensic psychiatry, uptake in practice is low. To overcome this problem, better integration of eHealth in treatment is necessary. In this perspective paper, we describe how the possibilities of eHealth can be connected to the risk-need-responsivity (RNR) model. To account for the risk-principle, stand-alone eHealth interventions might be used to offer more intensive treatment to high-risk offenders. The need-principle can be addressed by connecting novel experience-based interventions such as VR and apps to stable and acute dynamic risk factors. Finally, using and combining personalized interventions is in line with the responsivity-principle. Based on research inside and outside of forensic psychiatry, we conclude that there are many possibilities for eHealth to improve treatment—not just based on RNR, but also on other models. However, there is a pressing need for more development, implementation and evaluation research.

## Introduction

Forensic psychiatry is focused on treatment of people who display aggressive or sexual delinquent behavior that led or could lead to offenses and who simultaneously suffer from at least one psychiatric disorder, for example schizophrenia, antisocial personality disorder or post-traumatic stress-disorder (1–3). Treatment is offered to both out- and inpatients (4). Regardless of differences between levels of security, the main goal is to prevent (re)offending and thus to protect society. A meta-analysis has shown that 50% of offenders who did not receive treatment reoffend, as opposed to 38.8% of those who received psychological treatment (5). While these results are quite positive, recidivism rates show room for improvement. Cognitive behavioral therapy, a much-used form of psychological treatment, has been helpful in reducing recidivism, but has not been as effective for treatment of aggression as it is for treatment of anxiety or depression (5–7). There are multiple explanations for this. First, a pitfall of current treatment is that most forensic psychiatric patients are not that motivated for their often mandatory treatment (8, 9). Second, many forensic psychiatric patients experience difficulties with reflecting on their own behavior and emotions, which is an important skill for psychological treatment (10–12). Third, the forensic psychiatric patient population is extremely heterogeneous: there is no “typical” forensic psychiatric patient due to a large diversity in type of offense, mental disorders and socio-demographic background (13).

If developed and implemented well, technologies such as mobile apps, online modules, virtual reality (VR), serious games or wearables can be used to overcome some of the aforementioned barriers (14). The use of technology to support health, well-being and healthcare is referred to as eHealth (15). Technologies such as websites or mobile apps can be used to offer (parts of) existing treatment to a patient, enabling them to work independently on digital assignments or receive psycho-education *via* videos or written text (16). Immersive technologies such as VR can be used to transport patients to digital yet realistic environments in which they can practice with difficult situations and increase their coping skills (17, 18). Apps and wearables offer the possibility to collect contextual information from patients that cannot be retrieved in treatment rooms. Examples are wearables that continuously collect data on physiological signals such as heartrate variability or skin conductance, or experience sampling apps in which patients are asked about their experiences throughout the day (19). While there are more possibilities of technology, these examples illustrate that eHealth might have the potential to improve forensic mental healthcare (14).

However, there is a gap between the current situation and the potential of eHealth: uptake in practice is lagging behind on expectations (14, 17). An explanation for this gap is that eHealth is often used as a separate addition instead of in a blended way (20). Blended care refers to the combination of “offline,” in-person treatment with “online” technologies (21). By integrating offline and online care, it might be possible to combine the best of both worlds: offering new and unique types of treatment, while maintaining the advantages of the therapeutic alliance (22–24). A possible way to offer blended care and thus better embed technology in treatment is by integrating eHealth in models that are used to shape treatment.

In forensic psychiatry, the predominant, most-used treatment model to shape assessment and treatment of offenders is the Risk-Need-Responsivity (RNR) model (25, 26). The RNR model is based on three main principles: risk, need and responsivity. According to the risk principle, offenders that pose a high risk for reoffending should receive more intense levels of treatment. The need principle focuses on assessing and targeting criminogenic needs, also known as risk factors. In order to prevent offending, dynamic risk factors are especially relevant since these are changeable by means of specific interventions (27, 28). Finally, the responsivity principle of the RNR model prescribes that evidence-based interventions should fit the attributes of the individual offender, such as motivation or cognitive abilities.

In this perspective paper, we describe how the possibilities of eHealth interventions can be connected to the three main principles of the RNR-model to show how eHealth can be better integrated in treatment of offenders. By combining literature from both in- and outside of forensic psychiatry, we aim to identify directions to further improve treatment of offenders by means of technology. While this paper is structured by the RNR-model, the points that are raised are also relevant for other types of forensic treatment.

## eHealth and the Risk-Principle

The risk principle of the RNR model prescribes that the intensity of treatment should be adapted to the level of risk that a specific patient poses, which implies that high-risk patients require more intensive and frequent treatment than those that show a lower risk on re-offending and committing severe crimes. However, this is easier said than done, especially considering the increasing number of forensic psychiatric patients, combined with a shortage of staff and budget cuts (29–31). Web-based modules offer the opportunity to deliver treatment to patients regardless of time, place and availability of staff (15, 32). Systematic reviews on internet-based cognitive behavior therapy for multiple types of psychiatric disorders indicate that outcomes are comparable to face-to-face treatment (33, 34). The same trend seems to be recognizable in research on web-based intervention in forensic psychiatry: a review identified nine studies that focused on evaluation of different types of text-based digital interventions (14). Seven of those showed outcomes at least as effective as comparison groups and more effective than no intervention groups (16, 35–40), while only two studies found no improvements (41, 42). Consequently, psycho-education or standardized assignments in face-to-face treatment might be replaced by eHealth interventions, which could take valuable time of therapists' hands and provide them with more room for in-depth treatment. However, while they have the potential to make care more efficient, these interventions are hardly used in forensic practice, there have been questions about the fit of these mostly language-driven interventions with a low literacy target group, and their fit with the risk principle has not been investigated (43, 44). Additionally, inpatients are often not able or allowed to use technologies with internet access; either because this is policy of an inpatient clinic, or because the offense was related to the internet (44). Consequently, if eHealth is to be used to offer more treatment to high-risk patients, more attention needs to be paid to implementation, which is currently underrepresented in forensic practice and research (43–45).

## eHealth and the Need Principle

The need-principle of the RNR-model states that treatment should focus on reducing the dynamic risk factors of individual patients by means of targeted interventions (26). A distinction can be made between unchangeable static risk factors such as prior offenses or job history, stable dynamic risk factors such as antisocial attitudes, substance abuse, financial problems and antisocial associates, and acute dynamic risk factors, like access to a victim, exposure to drugs or a fit of rage (27, 46, 47).

### Stable Dynamic Risk Factors

There are multiple ways in which eHealth can be used to target stable dynamic risk factors. A first example is the use of technology to improve behavioral skills that are related to offending. Amongst other things, online modules or (secure) social networking sites can support patients in acquiring knowledge and skills about offense-related behavior, such as drug refusal skills (14, 35, 48). Furthermore, VR offers opportunities to actually practice with behavior in realistic contexts. To illustrate, a study outside of forensic psychiatry has shown that social skills training in VR led to increased conversational skills and assertiveness in psychiatric inpatients with schizophrenia, compared to regular social skills training (49). These types of studies highlight the potential of roleplaying in VR to support patients in improving important skills related to risk factors, such as emotion regulation or social functioning.

A second example is the use of technology to increase coping skills. A recent pilot study on an 8-week mindfulness training program showed a significant decrease in stress with an effect size of 0.39 directly in 13 forensic inpatients directly after the intervention. While no significant effects were found after a 3-month follow-up, this pilot study indicates that mindfulness might be a suitable coping method (50). Because a meta-analysis has shown that online mindfulness interventions are effective in general (51), mindfulness apps might be useful for forensic psychiatric patients as well. Another example is DEEP, an applied game that teaches diaphragmatic breathing in a gamified VR-environment *via* biofeedback. Although the effect was small, after using DEEP, anxiety was reduced in children in special education (52). These types of engaging, experience-based approaches might also be a good fit for enhancing relaxation skills in the often unmotivated forensic psychiatric patient populations (44).

Third, eHealth can be used to bolster self-control, which is one of the strongest protective factors and correlates of crime (53). Research has shown that heart rate variability (HRV) and skin conductance rise in the 20 min preceding aggressive outbursts of forensic psychiatric inpatients, which indicates that self-control failure might be predicted by changes in physiological variables (54). Consequently, wearables that provide direct feedback on HRV can offer “just-in-time coaching” and increase interoceptive awareness, which could in turn support patients in recognizing when coping strategies should be used to prevent self-control loss (55). Furthermore, a recent randomized controlled trial on interactive VR in forensic psychiatric inpatients showed no direct effects of VR on aggression, but regardless, several interesting findings emerged. Amongst other things, self-reported impulsiveness improved directly after the VR intervention compared to a control group, but this effect was not maintained on the long term (18). Even though these findings are not as convincing as expected, they do illustrate the potential of VR to target risk factors in a patient population whose behavior and treatment motivation are generally hard to improve. Finally, a self-control training app, in which participants train their “self-control muscle” by using their non-dominant hand for daily tasks, has been shown to be a promising way to target the automatic aspect of self-control in students, and might also be useful for forensic psychiatric patients (56).

Fourth, eHealth offers novel ways to target addiction, another important risk factor for offending. Besides existing self-help modules or apps for addiction (57), there are other ways to target addiction in hard-to-involve target groups. An example is the use of a gamified app based on evidence-based alcohol avoidance training (AAT), in which the user has to push away pictures of alcoholic beverages and pull non-alcoholic beverages toward themselves (58, 59). A pilot study with non-clinical sample with drinking problems showed promising results, amongst which a significant reduction in alcohol consumption after 3 months (59). This approach offers ways to also involve the automatic part of behavior in treatment of offenders. When using these types technologies, attention should not only be paid to their added value for individual patients, but also to ethical and privacy-related matters, such as ownership of data and the extent to which patients can and should be angered when immersed in offense-related VR scenarios.

While there are many possibilities, the connection between eHealth interventions and dynamic risk factors is not yet present in practice and research (17, 44). Consequently, it is important to explore and evaluate if and how eHealth can target risk factors, and to integrate this approach into clinical practice. In order to structure this, risk assessment instruments such as the FARE, HCR-20 or HCT-R can be of assistance (60–62). By creating an overview of eHealth interventions that can be used to target specific risk factors, the current “*ad-hoc*” approach - in which interventions are mostly selected because of their availability - can be overcome since therapists can select the most appropriate options for a patient's risk factors (43, 44). In Table 1, this is illustrated by means of examples.

**Table 1:** An overview of how different types of eHealth interventions can fit four of the risk factors of the HCR-20 (28, 63).

**Dynamic risk factor**	**Two examples of eHealth interventions**
Recent problems with insight	•Offense chain: multimodal online module to increase insight in what went wrong regarding the offense, with information, stories of forensic psychiatric patients and assignments with feedback (63). •Self-scoring app: mobile app in which the patient can assess their own protective and risk factors based on the HKT-R and HCR-20V3 to increase insight in risk factors (64)
Recent problems with instability	•Mobile app and biofeedback: chest strap to measure arousal and mobile app with just-in-time coaching based on physiological signals to prevent reactive aggression (55, 65) •Virtual reality aggression prevention training (VRAPT): Interactive VR for aggression regulation by means of roleplaying in virtual environments (18)
Recent problems with violent ideation or intent	•Virtual reality game for aggressive impulse management (VR-GAIME): serious game in VR that addresses bias toward aggressive facial expressions *via* gamified approach-avoidance training (66) •Aggression: multimodal online module to better deal with aggressive impulses by providing insight into thinking patterns, risky situations and pro's and cons of aggressive behavior (67).
Future problems with stress or coping	•DEEP: VR-game in which diaphragmatic breathing can be improved *via* biofeedback using a chest strap (68) •Diary moment of stress: mobile diary app with experience sampling to gain insight *in situations* or experiences that are related with experiencing stress (69).

### Acute Dynamic Risk Factors

While static and stable dynamic risk factors are incorporated in standardized risk assessment instruments, this is more challenging for acute dynamic risk factors for offending. Because these contextual factors - that occur directly before offending - are highly individual and only relevant for short periods of time and in specific situations, they are hard to identify and to improve in standard treatment, which is mostly based on talking and reflecting (70, 71). Identifying these factors in treatment requires fairly high levels of reflective skills, memory and honesty from forensic psychiatric patients (70, 71). Technologies offer multiple ways to identify and treat acute dynamic risk factors. One way to achieve this is by means of VR. In an interview study, therapists indicated that a possibility of VR might be that patients can be exposed to a broad range of personalized scenarios and in that way, insight might be gained into what “triggers” a patient when it actually occurs, as opposed to retrospectively talking about it, and targeted interventions and coping skills can be introduced (72) Furthermore, experience sampling *via* mobile apps might be used to gain more insight what triggers the patient throughout their daily lives. This could be asked at predetermined times, or when a specific event occurs, for example when a patient's heart rate variability (HRV) rises above the threshold value (19). While there are many possibilities to target triggers, it is not yet studied and integrated in clinical practice, so more research on this topic is required.

## eHealth and the Responsivity Principle

Finally, the responsivity-principle of the RNR-model states that the entire treatment should be tailored to fit the characteristics of forensic psychiatric patients (26). On the one hand, eHealth offers multiple ways to further integrate this principle in treatment, and on the other hand, the responsivity-principle should also be integrated in eHealth interventions. The one-size-fits-all approach toward eHealth in treatment of offenders is still predominant (14), while scientific research and experiences from clinical practice both highlight the need for personalized eHealth interventions, i.e., more responsive interventions (17, 72). For example, a study showed that the condition in which three additional computerized treatment sessions that were tailored to the individual of perpetrators of domestic violence was more effective than a non-tailored intervention (16). Research into the needs of therapists and patients also displayed the importance of personalized, tailored eHealth interventions because of the diversity and heterogeneity of the forensic psychiatric patient population (72).

eHealth can make treatment more responsive if interventions are successfully matched to the characteristics and preferences of patients. To illustrate: therapists indicate that patients who have difficulties with talking about their offense due to shame might benefit from written assignments in online modules (43, 44). Furthermore, new insights about risk factors that are generated by technologies such as VR or wearables can be used to better tailor treatment more to the needs of the patient (14, 44). Ideally, different types of eHealth interventions are combined to optimally fit the treatment of a patient: by integrating data from different technologies, a fuller picture can be painted (44). However, using eHealth in such a responsive way requires specific types of skills of therapists, highlighting the need for fitting education and training (44, 73). In order to unlock the potential of eHealth in forensic psychiatry, necessary preconditions seem to be that therapists have the necessary knowledge and skills required for using eHealth in a responsive way, and that they have an adaptive and flexible mindset toward experimenting with different technologies (74, 75).

## Discussion and Conclusion

Based on insights from research and practice in- and outside of forensic psychiatry, we provided some initial views on how eHealth can be integrated in the RNR Model. While it is obvious that more research is needed to further investigate effectiveness, eHealth interventions might be used to increase intensity of treatment of high-risk offenders by allowing them to independently work on parts of treatment. Furthermore, eHealth interventions such as VR and apps can offer new ways to identify and treat stable and acute dynamic risk factors. Finally, eHealth interventions should be more responsive, and the most optimal combination of interventions should be identified for individual patients. However, most of the possibilities are still potential and not used in practice and outcomes of evaluation studies are not always very convincing, so there is a pressing need for more research into if, how and when eHealth interventions are of added value for forensic mental healthcare.

In order to integrate eHealth in treatment guided by the RNR model, thorough development, implementation and evaluation are important (76). Multiple researchers recommended to develop new interventions *via* a participatory development approach, in which patients, therapists and other stakeholders are actively involved throughout the entire process (77–79). This approach is used to ensure that the intervention fits the characteristics, wishes, treatment protocols and risk factors (78). It is important to note that existing interventions from regular mental healthcare cannot simply be copy-pasted into this complex and unique setting, so they might have to be re-designed to optimally fit forensic psychiatry (14).

Second, thorough implementation in forensic organizations is a necessary precondition to achieve added value of eHealth. Despite its importance, implementation is underrepresented in both research and practice (44, 45). According to implementation models, attention needs to be paid to the required skills and attitudes of healthcare providers and patients, characteristics of the organization, demands of the wider context and their fit with the technology (43, 80). It seems that often, researchers, practitioners and management underestimate the importance of eHealth implementation. Consequently, factors from multiple levels need to be integrated in future research on implementation to account for all aspects of implementation (43).

Third, there is a need for more evaluation studies on eHealth in forensic psychiatric settings (14). To gain insight into effectiveness, classic randomized controlled trials can be used. However, depending on the research questions, other types of research designs that are more feasible and provide more insight into how and for whom eHealth works might be more suitable (81). Examples of methods that might be a good fit for these types of questions are single-case experimental designs, mixed-methods studies, realist evaluations or factorial designs (17, 76).

While this perspective paper provides some initial directions and insights into how eHealth can be integrated in the RNR model, it should be viewed as a starting point, and more research is required to further investigate how this should be achieved. In general, we suggest that more attention should be paid to eHealth interventions that can add something new that would not be possible in standard treatment, such as VR or wearables (76). Especially these types of interventions - which focus on doing and experiencing in a realistic context as opposed to thinking and talking in a treatment room - might be of added value for identifying and treating criminogenic needs in a responsive way (82). We also recommend that future work should not solely focus on benefits, but also on limitations of these types of interventions, including but not limited to barriers related to internet access, limited digital skills of patients and therapists, and ethical issues. To illustrate, a pitfall is that patients—especially when they receive treatment as part of a sentence—might feel that they must accept an eHealth intervention that is offered to them, while they might not feel comfortable with for example using a wearable due to privacy concerns. This highlights the importance of shared decision-making when shaping blended care—especially in those types of settings where patients have less autonomy. Finally, since the Good Lives Model, which applies a more positive psychology-approach, has been gaining ground (83, 84), future research could investigate how eHealth interventions fit within this model. A possible avenue to investigate is the way in which eHealth interventions can support offenders in reaching their “primary goods.”

To conclude: in order to ensure that eHealth can be of actual added value for forensic psychiatry, interventions need to be integrated in the predominant treatment model and have to be developed, implemented and evaluated by means of suitable research methods. In this perspective paper, we identified some ways in which the characteristics of eHealth interventions and the RNR model can be linked and provided multiple directions for future research and activities in practice. Finally, while for evidence-based practice, more research is obviously needed, we also might need to be aware that this does not become a reason - or excuse - not to try to innovate treatment by means of technology: at times it might even be helpful to just “take a leap of faith” and to challenge therapists and patients to start experimenting and trying out different types of eHealth interventions to determine what might be of added value. Based on our viewpoint paper, the most important theme seems to be a good fit: not just between eHealth and the RNR model, but also between technology, patient, therapist and treatment context.

## Data Availability Statement

The original contributions presented in the study are included in the article/supplementary material, further inquiries can be directed to the corresponding author.

## Author Contributions

HK drafted the first version of the paper. YB revised the paper. All authors accepted the final version of the paper.

## Conflict of Interest

The authors declare that the research was conducted in the absence of any commercial or financial relationships that could be construed as a potential conflict of interest.

## Publisher's Note

All claims expressed in this article are solely those of the authors and do not necessarily represent those of their affiliated organizations, or those of the publisher, the editors and the reviewers. Any product that may be evaluated in this article, or claim that may be made by its manufacturer, is not guaranteed or endorsed by the publisher.
